# Where neurostimulation meets neurodegeneration in Parkinson's disease related to GBA variants

**DOI:** 10.1002/acn3.52012

**Published:** 2024-03-26

**Authors:** Ludy C Shih, Margaret O'Connor

**Affiliations:** ^1^ Department of Neurology Boston Medical Center Boston Massachusetts USA; ^2^ Department of Neurology Chobanian and Avedisian Boston University School of Medicine Boston Massachusetts USA; ^3^ Department of Neurology Harvard Medical School Boston Massachusetts USA

Deep brain stimulation (DBS) for treating symptoms of moderate to advanced Parkinson's disease (PD) offers clinical benefit for tremor, motor fluctuations, and dyskinesias, but it may be associated with subtle changes in cognition. Given the prevalence of cognitive decline in people with PD,^1^ it is important to consider the additional cognitive effects of DBS; hence, preoperative neuropsychological evaluation has become standard in the evaluation of DBS candidacy. Recent studies have demonstrated that specific genetic risk factors, such as mutations in the gene encoding glucocerebrosidase (GBA), raise the risk for cognitive decline in PD.^2^, ^3^ Importantly, Pal and colleagues, in an international multicenter cohort, demonstrated clear differences in global cognition over time between groups of GBA mutation‐positive or mutation‐negative individuals who either did or did not have subthalamic nucleus (STN) DBS.^4^ Given what appears at first glance to be a decidedly negative outlook, how should clinicians convey what changes such an individual might expect if they proceed with DBS?

In this publication, Almelegy et al.^5^ add new data by examining cognitive performance in specific domains, namely response inhibition, episodic memory, and processing speed in a prospective cross‐sectional study of individuals with PD who underwent STN DBS from 2016 to 2023. Participants were matched for relevant clinical covariates, except for overall cognitive performance. Findings revealed that individuals who were GBA mutation‐positive and who underwent STN DBS (*n* = 9) had significantly worse performance on response inhibition as measured by the Flanker test, compared to a GBA mutation‐negative/STN DBS group (*n* = 17) or those who had GBA mutations but who did not have DBS (*n* = 14). There were no between‐group differences with respect to performance on tasks of episodic memory or processing speed, at an average of 2 years after DBS was initiated.

How might these findings contribute to our ability to predict clinically relevant cognitive change in these individuals over time? There are at least two questions here: (1) are the cognitive processes predictive of clinically significant symptoms, and (2) what length of follow‐up will be required to generate the data that changes clinical practice? The Flanker task reliably measures the ability to suppress responses to distractors and is considered a measure of executive function. Poorer performance has been seen in individuals with PD, but individual items like the Flanker task performance by themselves may not be strong drivers of overall cognitive function. As such, we await additional data on change in the other cognitive domains over time in this cohort.

Second, what length of time might it require before we see appreciable differences between mutation‐positive and mutation‐negative individuals, with and without DBS? A minimum of 36 months was studied in the Weaver et al.^6^ and Boel et al.^7^ studies, both of which also suffered from dropout in the long‐term observation period. This reality also begs the question—what is a minimally important cost difference in comparison to potential benefit of DBS with respect to physical discomfort from motor complications? Longitudinal data, ideally from a parallel‐arm study with patients randomized assignment to surgery or best medical therapy would give us the gold standard, but practicalities make such an ideal scenario unlikely to happen. A much earlier clinical study at Queen Square Hospital in the United Kingdom revealed clear differences in global cognition between individuals with PD with GBA risk variants versus mutation‐negative patients.^8^ Over a 10‐year period, only 16 individuals with GBA risk variants and mutations were recruited. It is safe to assume that even in large urban academic centers performing over 50–100 new DBS electrode implantations a year, there are likely only a handful of individuals with a GBA mutation seeking advice regarding DBS in a given year.

On this literally case‐by‐case basis, it now becomes apparent that advice based upon emerging published data needs to be translated cautiously, helping the patient and their care partners consider their own personal and social circumstances and the number of quality years they may have to enjoy with only mild to moderate disability, data notwithstanding. Furthermore, we still do not know whether the globus pallidus internus (GPi) target may confer less risk for cognitive decline, irrespective of mutation status.^6^, ^7^ Because of the uncertainty and the need to take into account factors that may not translate well to the research setting, it seems more important than ever to enable shared decision‐making and support for patients, however they may choose.
